# Tracheoinnominate fistula: acute bleeding and hypovolemic shock due to a trachea-innominate artery fistula after long-term tracheostomy, treated with a stent-graft

**DOI:** 10.1186/s42155-021-00216-8

**Published:** 2021-03-19

**Authors:** Ali Khanafer, Victoria Hellstern, Helfried Meißner, Christoph Harmening, Klaus Schneider, Hans Henkes

**Affiliations:** 1 Neuroradiologische Klinik, Klinikum Stuttgart, Kriegsbergstrasse 60, 70174 Stuttgart, Germany; 2 Klinik für Gefäßchirurgie, Endovaskuläre Chirurgie und Transplantationschirurgie, Klinikum Stuttgart, Kriegsbergstraße 60, 70174 Stuttgart, Germany; 3 Klinik für Anästhesiologie, Operative Intensivmedizin, Notfallmedizin und Schmerztherapie, Klinikum Stuttgart, Kriegsbergstraße 60, 70174 Stuttgart, Germany; 4 Klinik für Hals-, Nasen-, Ohrenkrankheiten, Plastische Operationen, Klinikum Stuttgart, Kriegsbergstraße 60, 70174 Stuttgart, Germany; 5 Medical Faculty, University Duisburg-Essen, Essen, Germany

**Keywords:** Tracheostomy, Tracheoinnominate fistula, Stent-graft

## Abstract

**Background:**

A tracheo-innominate fistula is a rare but life-threatening complication of tracheostomy and has a mortality rate of 100% without therapy. The underlying cause is an acquired fistula between the brachiocephalic trunk and the trachea, induced by a tracheostomy cannula’s mechanical impact.

**Case presentation:**

A 25-year-old female was admitted with pulsatile bleeding from a tracheostomy. The cause of the bleeding was a tracheo-innominate artery fistula, which was difficult to recognize. Said fistula was treated with implantation of a self-expanding stent-graft. The bleeding stopped immediately after the implantation of the stent-graft. Dual antiplatelet medication with aspirin IV and ticagrelor PO, bridged with a bolus of eptifibatide IV, was started right after the stent deployment.

**Conclusions:**

Endovascular self-expanding stent-graft implantation is a viable treatment option for tracheo-innominate artery fistulae, especially in hemorrhagic emergencies.

## Background

Tracheo-innominate fistula (TIF) is a sporadic (0.1%–1%) and potentially lethal complication after tracheostomy (Cooper and Grillo 1969; Qureshi 2018). In most cases (80%), the fistula is located between the brachiocephalic trunk (“innominate artery”) and the anterior aspect of the trachea. TIF mostly develop in a period of 3 days to 6 weeks after tracheostomy. The clinical manifestation is sudden massive tracheal hemorrhage. Rapid management is essential during the acute stage due to life-threatening airway obstruction and hemorrhagic shock. TIF has a high mortality rate, reaching 100% in the absence of treatment. Open surgery is the traditional treatment for TIF. However, endovascular stenting and embolization have become alternative treatments in recent years (Taechariyakul et al. 2020). We report a case of a patient with TIF that was successfully treated with endovascular stent graft reconstruction of the innominate artery.

## Case presentation

A 25-year-old female was brought to our Emergency Room with acute pulsatile bleeding from her tracheostomy with an 8.1 mg/dl hemoglobin value upon admission. The patient suffered from long-term spastic tetraparesis due to cerebral palsy. Sixty months ago, the patient had undergone tracheostomy as a treatment of respiratory failure after aspiration pneumonia with sepsis. Inspection of the trachea using a flexible bronchoscope demonstrated active arterial bleeding from the trachea anterior wall with pooling of blood in the dependent airway. Blood was aspirated to clear the airways, and the cuff of the tracheostomy tube (Tracheoflex 8 mm, Teleflex Medical) was overinflated to control the bleeding in the short term. A diagnostic digital subtraction angiography (DSA) was carried out under emergency circumstances. The initial angiogram with the cuff inflated revealed no visible contrast medium extravasation. Therefore, the cuff of the tracheal tube was first deflated and then the tube was temporarily removed.

General anesthesia was induced with propofol and sufentanil, neuromuscular blockade was achieved by atracurium. Maintenance was provided with continuous infusion of propofol and remifentanil. A 9F sheath (Terumo) was inserted into the right common femoral artery. A diagnostic digital subtraction angiography (DSA) was carried out using a Tempo4 vertebral 4F catheter (Codman) under emergency circumstances. Straight posterior-anterior and 20° right and left anterior oblique angiographic projections of the brachiocephalic artery with 6 frames per second were acquired with manual bolus injection of each 8 ml Imeron 300 (Bracco Imaging).

**Fig. 1:**
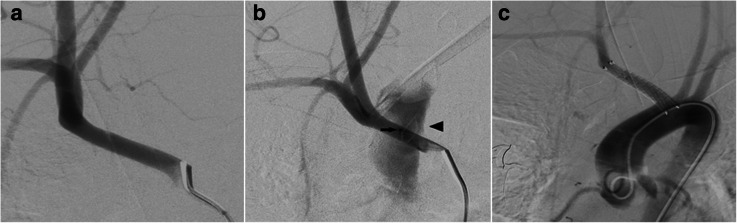
Diagnostic imaging in a 25-year-old female patient with a tracheoinnominate fistula. DSA of the brachiocephalic artery in posterior-anterior projection with an overinflated tracheal tube showed no bleeding source (**a**). Temporary removal of the tracheal tube and releasing the cuff revealed the contrast medium extravasation from the brachiocephalic artery’s posterior wall to the anterior wall of the adjacent trachea, with contrast medium in the trachea (arrow: bleeding spot; arrowhead: contrast medium in the trachea (**b**). The following completion flush DSA run of the aortic arch in a left anterior oblique projection showed the proper wall apposition of the stent-graft, the interruption of the previous extravasation, and the patency of the origins of the right subclavian and both common carotid arteries. The stent-graft was reaching slightly too far proximal into the aortic arch but did not compromise the origin of the left common carotid artery (**c**)

The acquisition of an aortogram was considered but we decided to upfront continue with selective injections of the brachiocephalic trunk. The brachiocephalic trunk’s selective injection demonstrated a difficult to recognize small dot of contrast medium accumulation, which was considered the site of extravasation from the posterior wall of the brachiocephalic trunk to the anterior aspect of the adjacent trachea. We decided to implant a self-expanding stent-graft into the brachiocephalic trunk to seal the vessel wall’s erosion without covering the subclavian artery’s origin. Using the inbuild measurement tool of the DSA machine (Siemens Axiom Artis Zee) with a calibration of the distance between the target vessel and the table surface, the diameter and length of the brachiocephalic artery was determined as 6.9 mm and 36.7 mm, respectively. The available implant was a Fluency Plus Stent Graft 8 mm × 40 mm (BD Bard), mounted in an 80 cm long catheter with a 9F crossing profile. A stiff 0.035″ diameter and 300 cm length Radiofocus guidewire (Terumo) with an angled tip was navigated into the right external carotid artery using a 4F Tempo4 vertebral catheter (Codman) through a 9F sheath (Terumo), followed by the removal of the 4F catheter. The Fluency Stent Graft was inserted over the said guidewire into the brachiocephalic trunk without using a guide catheter. Deployment of the stent-graft immediately stopped the tracheal bleeding (Fig. 1). Post-deployment adaptation of the stent-graft to the vessel wall with balloon angioplasty was not required, since the stent-graft instantaneously interrupted the extravasation. The implanted stent-graft was slightly longer than desired. The proximal end was reaching into the aortic arch but did not compromise the origin of the left common carotid artery. To prevent a thromboembolic occlusion of the stent, a loading dose of 1× 500 mg acetylsalicylic acid (ASA; Aspirin, Bayer Vital) IV and 1× 9 mg eptifibatide (Integrilin, GlaxoSmithKline) IV were given before implantation; 1× 180 mg ticagrelor (Brilique, AstraZeneca) PO was administered immediately after access via a feeding tube. The final DSA run demonstrated no active bleeding, exclusion of the fistula and flow in the right internal carotid, subclavian and vertebral arteries. Daily medication consisted of 2× 500 mg ASA IV and 2× 90 mg ticagrelor PO. Daily testing with Multiplate (Roche Diagnostics) and VerifyNow (Accriva) confirmed dual platelet function inhibition. A follow-up cranial CT was obtained after 30 days due to fluctuating vigilance and revealed no ischemic cerebral lesions. Follow-up bronchoscopy performed after 17 days for recurrent pneumonia showed no bleeding. Antibiotic treatment with Piperacillin/Tazobactam 4 g/0,5 g IV (HEXAL) was initiated on day 1 after the endovascular procedure. After discharge on day 8 after the procedure, the patient suffered from complicated pneumonia. During the second clinical admission, she died 31 days after the endovascular treatment in the intensive care unit from pulmonary failure.

## Discussion

Long-term tracheostomy may cause a fistula’s development between the trachea and the brachiocephalic trunk due to tracheitis, focal necrosis, cartilage loss, and then fistulization (Komatsu et al. 2013). Common risk factors are low tracheostomy position, excessive cuff inflation, and steroids (Qureshi 2018). The rapid management and the early control of the hemorrhage play a crucial role in patient survival (Komatsu et al. 2013; Seung et al. 2012). The primary goal is to secure the airway by hyperinflating the tracheal cuff and an endotracheal tube’s advancement.

Clinical signs of incipient TIF are small amounts of blood from the tracheostomy, hemoptysis, or pulsatile movements of the tracheal cannula (Schaefer and Irwin 1995). It can be challenging to confirm the diagnosis of TIF with computed tomography angiography and may require bronchoscopy and catheter angiography to determine the location of the bleeding (Seung et al. 2012). In a typical emergency, it is not feasible to establish a sophisticated work-up. A rapid diagnosis will allow the decision-making for an emergent therapy concept (Reger et al. 2018).

There are various techniques available for the treatment of acute TIF. The immediate surgical treatment is the most common approach to controlling bleeding and reconstructing the vessel and the tracheal defect (Komatsu et al. 2013). A median sternotomy is required to gain access. Once the TIF is identified, the vascular clamps are placed to stop the bleeding. The defect can be reconstructed by ligation or resection with replacement by a vascular prosthesis. Reconstruction with a bypass is also possible in emergencies. The mortality rate after emergency surgery has been reported to be beyond 50%. Neurological deficits of 10% and sternal wound complications of 39% were reported (Seung et al. 2012; Reger et al. 2018; Nakai et al. 2013).

Endovascular therapy is increasingly considered an attractive and practical approach to treat arterial bleeding due to fistulas and vascular erosions. There are few publications in the English literature discussing the endovascular treatment of TIF, either as a stand-alone treatment or as a bridging concept for patients in a poor clinical condition to operate on them in the subacute phase after at least partial recovery (Taechariyakul et al. 2020).

Taechariyakul et al. (2020) identified 261 published cases of TIF in a meta-analysis and reported a lower procedure-related complication rate (30% vs. 50%, *p* = 0.045) and 30-day mortality (9% vs. 23%, *p* = 0.008) for the endovascular treatment compared to surgery. Endovascular stent-graft implantation is a rapid and safe procedure. However, possible complications such as graft infection, graft occlusion, and postoperative rebleeding due to tracheal erosion must be considered (Nakai et al. 2013).

In our case, we opted for an endovascular therapy due to the high-risk of the already unstable patient and because open surgery was considered hazardous, especially with a vaguely identified bleeding source. In the English literature, reports of post-procedural complications such as occlusion of the right carotid artery or right subclavian artery leading to steal syndrome can be found. Therefore, the decision for the correct stent dimensions and position is crucial.

## Conclusions

Endovascular treatment of TIF with a stent graft is both effective and safe. The procedure is well suited in acute situations to avoid fatal exsanguination.

## Data Availability

The primary data underlying this manuscript are available from the corresponding author upon request.

## Abbreviations

CT: Computed tomography
DSA: Digital subtracted angiography
F: French catheter scale
